# Timings of attentional switching to perturbation and postural preparation during transient forward or backward floor translation

**DOI:** 10.1186/s40101-017-0162-6

**Published:** 2018-01-08

**Authors:** Katsuo Fujiwara, Chie Yaguchi, Maki Maekawa, Naoe Kiyota

**Affiliations:** 1 Department of Sports and Health, Kanazawa Gakuin University, 10 Sue-machi, Kanazawa, 920-1392 Japan; 2 Department of Rehabilitation, Japan Health Care College, 6-17-3 Megumino-nishi, Eniwa, 061-1373 Japan; 3 Department of Physical Education, International Pacific University, 721 Kannonji, Seto-cho, Higashi-ku, Okayama, 709-0863 Japan

**Keywords:** Attentional switching, Postural preparation, Transient floor translation, Event-related brain potential, Electromyogram, Center of pressure

## Abstract

**Background:**

Relationships between the timings of attentional switching and postural preparation were investigated using a choice-reaction paradigm with transient floor translation (S2), with the direction indicated by a warning auditory signal (S1).

**Methods:**

Thirteen healthy young adults participated in this study. S2 started 2 s after S1 onset while standing on the platform. The platform moved forward when S1 was a high tone, and backward when S1 was a low tone. In the S1–S2 period, attentional switching was evaluated by P3 component of event-related potential.

**Results:**

A shift in the center of pressure in the anteroposterior direction (CoPap) or a continuous increase in postural muscle activation toward S2 was recognized as postural preparation. Changes in postural muscle activation were found just before the CoPap shift. P3 was observed about 250–650 ms after S1. Onset of postural preparation was significantly later (about 200 ms) than latency of P3 (*p* < 0.001) and correlated strongly with P3 latency (forward: *r* = 0.81, backward: *r* = 0.74, *p* < 0.01).

**Conclusion:**

Postural preparation for S2 was demonstrated to start after attentional switching from S1 to S2.

## Background

Rapid attentional switching from sensory information and/or an ongoing task to a postural disturbance is an important function for postural control [1, 2]. Particularly in the elderly, reduced ability in attentional switching has been suggested as an important factor in age-related deterioration of balance control [1]. To investigate attentional switching, previous studies have used cognitive and postural control tasks that were conducted simultaneously, and analyzed changes in performance of these tasks [1, 3, 4]. However, few studies have used neurophysiological methods [5, 6].

We have investigated attentional allocation directed to postural control associated with floor translations, using event-related brain potential (ERP) [7, 8]. Contingent negative variation (CNV) of ERP, induced by an S1 (warning signal)–S2 (response signal) paradigm [9], could evaluate anticipatory attention directed to S2 [10, 11]. The present study set a choice-reaction paradigm with transient floor translation (S2), where S2 direction changed forward or backward in accordance with the preceding S1. In this S1–S2 choice-reaction paradigm, S1 has roles in both indicating translation direction and acting as a warning signal. Attention should therefore be initially directed to S1 and then switched from S1 to S2, for appropriate preparation for S2. Attentional switching can be evaluated by the P3 component of ERP, as described below.

In such S1–S2 choice-reaction paradigms with finger flexion, a positive potential is observed at the parietal electrode about 400–600 ms after S1, in contrast to the paradigm with repetition of a fixed S2 task [12]. This positive potential, which some researchers regard as the P3 component [13, 14], is considered to reflect attentional switching to a task in response to S2 after task judgment at S1 [12–15]. P3 elicited in an oddball task [16] reflects cognitive processing, such as evaluation and judgment of sensory stimuli [17] and subsequent context updating [16, 18]. P3 tended to show bimodal peaks in a Stroop paradigm and a multiple-choice reaction task in which time was required for evaluation and judgment of stimulus [19, 20], while in a task with high time pressure, peaks overlapped, and consequently a unimodal peak [21] or a steeper slope of the P3 waveform after the first peak were seen [5]. These findings probably indicate that the first and latter parts of P3 waveform would reflect evaluation and judgment of sensory stimuli and attentional switching for task execution, respectively.

Backward and forward translations of the floor induce postural disturbances in the opposite direction. With either disturbance, preparatory muscle activation is observed between S1 and S2, which would relate to increased muscle stiffness or control of the standing position with the following displacement of the center of pressure in the anteroposterior direction (CoPap) [7]. As the activating muscles differ between forward and backward translations (frontal and dorsal muscles, respectively) [7, 8], postural preparation and attention may change in accordance with the direction of floor translation. In the present paradigm, postural preparation would thus be started after evaluation of S2 direction, or attentional switching from S1 to S2.

In this study, timing relationships between attentional switching and postural preparation were investigated, using the S1–S2 choice-reaction paradigm with transient forward or backward floor translation (S2), with the direction indicated by S1. The working hypotheses were that under this paradigm, P3 would be observed and preparatory muscle activation would start after the latter peak point of P3.

## Methods

### Subjects

Thirteen healthy young adults (6 men, 7 women) participated in this experiment. Mean (± standard deviation (SD)) age, height, weight, foot length (FL), and auditory threshold were 23.0 ± 5.0 years, 166.7 ± 10.0 cm, 59.3 ± 9.5 kg, 24.7 ± 1.8 cm, and 28.5 ± 3.8 dB, respectively. No subject had any history of neurological or orthopedic impairment. Informed consent was obtained from all subjects following an explanation of the experimental protocol, which was approved by the ethics committee at Kanazawa University.

### Apparatus and data recording

**Fig. 1:**
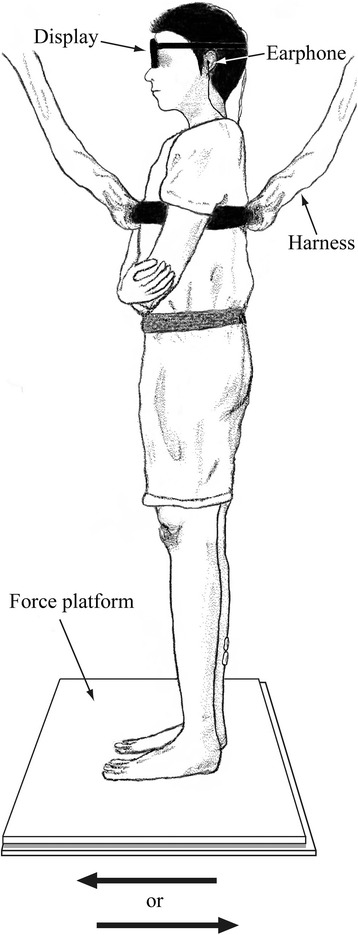
Experimental setup

A force platform (FPA34; Electro-design, Japan) was used to measure CoPap (Fig. 1). Electronic signals for CoPap were sent simultaneously to one computer (PC9801BX2; NEC, Japan) to determine CoPap position online, and to another computer (Dimension E521; Dell Japan, Japan) for analysis. The former received CoPap data via an A/D converter (PIO9045; I/O-Data, Japan) at 20 Hz with 12-bit resolution and could generate a buzzer sound when the CoPap was located within ± 1 cm of the required position. CoPap position was calculated and represented as the percentage distance from the heel in relation to FL (%FL).

The platform was fixed to a handmade table that was horizontally movable in an anteroposterior direction by a linear motion guide actuator (SKR4610A-0290-1-1001; THK, Japan) with a computer-controlled electric motor (SANMOTION model No. PB PBBR604; Sanyo Denki, Japan). The direction, velocity, amplitude, and onset timing of platform movement were adjusted by the motor (amplitude error: mean 0.5 (SD 0.06) cm; timing error: mean 1.1 (SD 2.2) ms). Floor translation was detected by an accelerometer (AS-2GB; Kyowa, Japan) fixed to the platform.

S1 was provided as two types of auditory stimulus delivered via earphones with intensity of 35 dB above the threshold and duration of 50 ms. S2 was a transient floor translation in which the direction was set as forward or backward when the frequency of S1 was 2000 or 1000 Hz, respectively.

Ag-AgCl cup electrodes (8-mm diameter) for recording electroencephalogram (EEG) were placed on the scalp at Fz, Cz, and Pz in accordance with the international 10-20 system, and referenced to linked ear lobes. A ground electrode was placed at Fpz. Electrooculogram (EOG) was recorded from a pair of electrodes placed above and below the right eye. To fix the eye position, subjects were instructed to gaze at a fixation point presented on an eye-trek face-mounted display (FMD011F; Olympus, Japan). Surface electrodes (P-00-S; Ambu, Denmark) were used in bipolar derivation to record surface electromyograms (EMGs) of the following muscles on the right side: rectus abdominis (RA), erector spinae (ES), rectus femoris (RF), biceps femoris (BF), tibialis anterior (TA), medial head of gastrocnemius (GcM), and soleus (Sol). For each muscle, electrodes were fixed after shaving and cleaning the skin with alcohol. Electrodes were aligned along the long axis of the muscle with an inter-electrode distance of about 3 cm. Electrode input impedance was < 5 kΩ. Signals from electrodes were amplified (EEG: × 40,000; EOG: × 4000; EMG: × 4000) and band-pass filtered (EEG: 0.05–100 Hz; EOG: 0.05–30 Hz; EMG: 5–500 Hz) using an amplifier (Biotop 6R12; NEC-Sanei, Japan).

For subsequent analyses, all electrical signals were sent to the computer for analysis via an A/D converter (ADA16-32/2(CB)F; Contec, Japan) at 1000 Hz with 16-bit resolution.

### Procedure

All measurements were carried out while the subject stood barefoot, with the feet 10 cm apart and parallel on the force platform, and the upper limbs crossed in front of the body (Fig. 1). These positions were set so as to achieve a relatively fixed postural control without use of the upper limbs for maintenance of balance during the experiment. To prevent falls due to floor translation, subjects wore a harness around the chest.

First, mean position of CoPap was measured while the subject maintained a quiet standing (QS) posture for 10 s. The mean value of the five trials was adopted as the QS position. Next, mean CoPap position during the extreme backward leaning (EBL) posture was measured twice. Subjects gradually leaned backward from QS for approximately 5 s, pivoting at the ankles with the rest of the body kept aligned, then maintained this EBL posture for 3 s. The more posterior CoPap mean position of the two trials was adopted as the EBL mean position, and the posterior peak position of CoPap in the adopted trial was defined as the EBL peak position. For the extreme forward leaning (EFL) posture, EFL mean position and forward peak position of CoPap were measured in the same manner as EBL posture.

Next, subjects performed floor translation tasks. Preceding the experimental session, velocity and amplitude of floor translation were set for each subject based on EBL mean and peak positions, as follows [8, 22]. To begin, a 5- or 10-cm floor translation was applied at a velocity of 20 cm/s. If the posterior peak of CoPap after the translation at either amplitude was located between EBL mean and peak positions, 20 cm/s was adopted as the translation velocity. If not, velocity was reduced or increased until the posterior peak at either amplitude was located between these positions (change in 5-cm/s increments). Second, a linear regression line was drawn through the two coordinates of floor translation amplitude (5 and 10 cm) and posterior peak of CoPap at the determined velocity. Based on the line, the translation amplitude was determined as the posterior peak located midway between the EBL mean and peak positions. Mean adopted translation velocity and amplitude were 18.1 ± 5.6 cm/s and 6.4 ± 2.2 cm, respectively. The same velocity and amplitude were used for forward and backward floor translation.

Experimental sessions comprised two simple-reaction tasks with forward or backward floor translation and one choice-reaction task. Simple-reaction tasks were undertaken to clarify the unique characteristics of P3 component in the choice-reaction task. In the simple-reaction task, the same S1 was presented in the session, and the direction of floor translation was fixed. In the choice-reaction task, either version of S1 was presented with 50% presentation probability in a random order in each trial, and the direction of S2 was determined according to S1. In both tasks, the subject maintained CoPap position within the QS position ± 1 cm until S2 onset. This range of QS positions was presented by the buzzer sound for at least 3 s before S1 onset. S1 was randomly presented 1–2 s after the experimenter stopped the buzzer sound, then S2 started 2 s after S1. Subjects were instructed to avoid changing the initial foot position in response to S2. In each task, a set of 10 trials was repeated until the number of trials accepted for EEG averaging exceeded 12 trials in each floor direction. No trials were seen in which foot position changed in response to S2. Trials were excluded if CoPap deviated over ± 1 cm from the QS position before S2. Moreover, trials with eye blinks or movement artifacts (voltage at EOG or any EEG electrode exceeding ± 100 μV) between 300 ms before S1 and S2 were excluded from EEG averaging.

The order of tasks was forward simple-reaction, backward simple-reaction and choice-reaction tasks. Before the experimental session, 5 practice trials were performed in each simple-reaction task. In the choice-reaction task, the judgment of floor translation by S1 was fully practiced in a seated posture; then, one practice trial of floor translation was performed. Subjects were given a standing rest period of 30 s between trials and a seated rest period of 3 and 5 min between each set and between each task, respectively.

### Data analysis

All data analyses were performed using BIMUTAS II software (Kissei Comtec, Japan). In the following analyses, the late component of contingent negative variation (late CNV) just before S2 and CoPap and EMG after S2 were analyzed for simple- and choice-reaction tasks to separately investigate differences in postural preparation and postural control at the S2 point between both tasks according to floor direction. P3, CoPap and EMG between S1 and S2 were evaluated for the choice-reaction task only to investigate the relationship between attentional switching and postural preparation.

For forward and backward floor translation in each task, to evaluate the magnitude of backward and forward disturbance induced by S2, the posterior and anterior peaks of CoPap after S2 were identified in each trial, and the distances from EBL and EFL mean position to this peak position were calculated, respectively. The mean value from all trials adopted for EEG averaging was defined as the CoPap displaced position.

All EMGs were 40-Hz high-pass-filtered to exclude electrocardiographic and movement artifacts, then full-wave-rectified. For the analyses of postural muscle activation after S2, frontal (RA, RF and TA) and dorsal (ES, BF, GcM and Sol) muscles were used for analysis with forward and backward floor translation, respectively. In each trial adopted for EEG averaging, the envelope line of the EMG burst, continuing for at least 50 ms, was identified by visual inspection of the EMG trace on a computer. Burst onset was defined as the point at which EMG deviated more than the mean + 2 SDs of the background activity during the standing posture before S1, and the time of onset compared to S2 was measured. EMG waveforms from − 300 ms to + 500 ms with respect to the burst onset were averaged separately for each direction in each task. Averaged waveforms were smoothed using a 40-Hz low-pass filter; then, a peak was identified [5, 8, 23]. The peak amplitude from baseline and the peak time with respect to burst onset were measured.

**Fig. 2:**
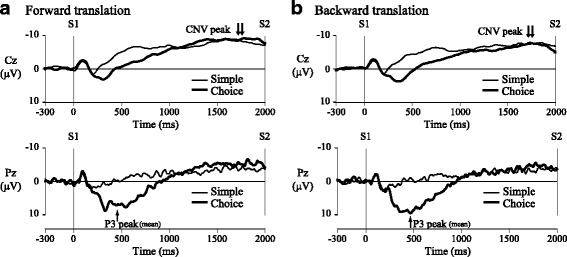
**a**, **b** Grand average waveforms of contingent negative variation (CNV) and P3 component

Waveforms of EEG from 300 ms before S1 to S2 were averaged for all trials adopted as EEG averaging separately for all combinations of floor direction and task. At least 14 trials were adopted for each averaging. The mean amplitude of the 300-ms period before S1 was used as a baseline for averaging EEG. Waveforms recorded from Cz, in which late CNV was maximal in all tasks, were used for CNV analyses (Fig. 2). Averaged waveforms of EEG at Cz were 4-Hz low-pass filtered. The maximal negative potential identified from 1400 ms after S1 to S2 was defined as the CNV peak [23], and latency relative to S2 and amplitude from the baseline were calculated as CNV peak time and amplitude, respectively. The P3 component was observed only in the choice-reaction task, and its amplitude was the largest in the parietal area (Fig. 2). As a result, P3 was analyzed in the waveform recorded from Pz. The averaged waveforms were smoothed using a 30-Hz low-pass filter. In many P3 waveforms, bimodal peaks were observed. Accordingly, P3 was analyzed as follows: first, the largest positive peak between 250 and 650 ms after S1 was defined as P3. Another P3 peak was defined as present when the second-largest positive peak observed around the largest P3 peak was larger than 70% of the largest peak amplitude, and when the amplitude of the trough between these two positive peaks was less than 95% of the second-largest peak amplitude [5]. These P3 peaks were referred to as the first and second peaks according to the latency. P3 latency was calculated as the interval from S1 to the second peak. If P3 was unimodal, the peak was adopted for P3 latency calculation.

**Fig. 3:**
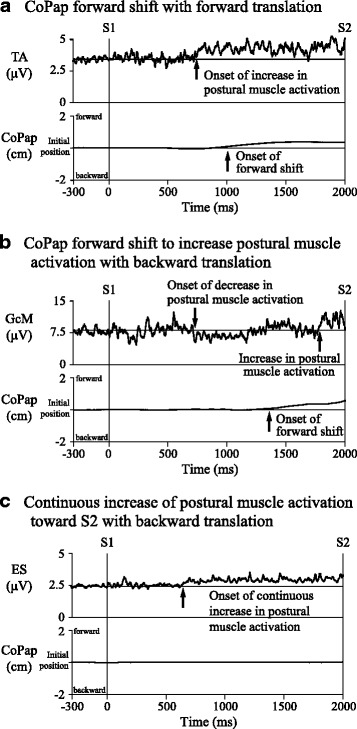
**a**–**c** Representative waveforms for electromyogram and center of pressure in the anteroposterior direction (CoPap) between S1 and S2 for subjects using different postural preparation strategies. TA: tibialis anterior; GcM: gastrocnemius; ES: erector spinae

Waveforms of rectified EMG and CoPap from 300 ms before S1 to S2 were averaged for all trials adopted as EEG averaging separately for floor direction. The averaged waveforms of EMG and CoPap were 30-Hz low-pass filtered (Fig. 3). In most EMG and CoPap waveforms, changes for postural preparation were observed in the early period of S1–S2. Thus, changes in EMG and CoPap as postural preparation were analyzed for the 1400 ms period after S1 when the late CNV peak was not present [23]. For CoPap, forward or backward shift was defined as CoPap deviation more than 2 SD above or below the mean CoPap amplitude in the 300-ms period before S1 (base period). When CoPap shifts were found, changes in EMG activation were followed by the start of the shift. Some subjects showed EMG changes without CoPap shift. Therefore, the onset of changes in postural muscles’ activation was defined as the time point at which activation deviated by more than 1 SD above or below the mean EMG amplitude in the base period. The onset time of preparatory muscle activation was defined as the time difference from S1. When several muscles showed such defined changes, the muscle with the largest ratio of change was adopted for analysis. To investigate differences between the timings of attentional switching and postural preparation, time differences were calculated between P3 latency and onset time of preparatory muscle activation.

### Statistical analysis

The Shapiro-Wilks test confirmed that all data satisfied the assumptions of normality. The paired *t* test was used separately for floor direction to evaluate task differences in time and amplitude of CNV peak between S1 and S2, and CoPap displaced position and onset time, peak time and peak amplitude of each EMG burst activity after S2. The following statistical analyses were performed only in the choice-reaction task. The paired *t* test was used to evaluate the difference in P3 latency between floor directions. For the onset time of preparatory muscle activation, and time difference between onset time and P3 latency, after Levene’s test was used to confirm whether the variance was equal, a non-paired *t* test was used to investigate differences between floor directions only for those subjects showing postural preparation. Pearson’s correlation was used separately for floor direction to evaluate the magnitude of correlation between the onset time of preparatory muscle activation and P3 latency. The alpha level was set at *p* < 0.05. All statistical analyses were performed using IBM SPSS Statistics 19 (IBM Japan, Japan).

## Results

In response to S2 for both directions, no significant differences between tasks were found in CoPap displaced position, onset time, peak time or peak amplitude of each postural muscle activation (Table 1). For both directions, no significant differences were identified between tasks in the time or amplitude of the CNV peak (Table 1).

**Table 1:** Means and standard deviations (SDs) of each parameter of contingent negative variation (CNV), electromyograms and center of pressure in the anteroposterior direction (CoPap)

Dependent variables		Forward translation			Backward translation			
S1–S2 period								
CNV peak time (ms)	Simple	317.2 ± 208.3			270.1 ± 198.8			
	Choice	326.3 ± 205.6			229.4 ± 142.9			
CNV peak amplitude (μV)	Simple	10.5 ± 4.4			9.9 ± 4.8			
	Choice	10.9 ± 5.7			9.4 ± 4.4			
After S2								
CoPap displaced position (%FL)	Simple	0.0 ± 6.4			− 7.0 ± 8.6			
	Choice	2.3 ± 5.5			− 8.4 ± 7.3			
		RA	RF	TA	ES	BF	GcM	Sol
Onset time of postural muscle (ms)	Simple	144.5 ± 15.0	109.5 ± 15.1	94.4 ± 11.6	121.5 ± 14.9	110.5 ± 16.4	90.2 ± 8.2	78.6 ± 12.1
	Choice	141.6 ± 15.5	109.2 ± 15.5	94.1 ± 12.1	120.5 ± 9.8	103.9 ± 11.2	88.2 ± 7.5	77.5 ± 8.3
Peak time of postural muscle (ms)	Simple	97.7 ± 115.7	90.5 ± 104.8	56.5 ± 18.5	68.2 ± 62.7	56.4 ± 30.6	34.2 ± 10.6	34.7 ± 18.5
	Choice	106.8 ± 132.4	78.7 ± 92.3	49.2 ± 16.8	59.4 ± 37.9	66.0 ± 46.9	35.6 ± 6.0	35.3 ± 21.5
Peak amplitude of postural muscle (μV)	Simple	21.8 ± 16.0	63.0 ± 40.7	319.7 ± 107.5	41.8 ± 27.5	31.2 ± 15.7	194.3 ± 96.0	95.9 ± 32.9
	Choice	16.1 ± 11.1	54.0 ± 31.3	290.5 ± 108.1	42.1 ± 21.3	33.7 ± 18.4	182.9 ± 84.7	86.4 ± 28.1

Mean ± SD

*RA* rectus abdominis, *RF* rectus femoris, *TA* tibialis anterior, *ES* erector spinae, *BF* biceps femoris, *GcM* medial head of gastrocnemius, *Sol* soleus, *FL* foot length

In the choice-reaction task only, a large positive peak (P3) was clearly found around 450 ms after S1 onset (Fig. 2). P3 latency was 448.4 ± 110.0 ms with forward translation and 452.5 ± 96.5 ms with backward translation. No significant difference in P3 latency was found between floor directions. In addition, the following postural preparations were observed between S1–S2 period in most subjects: (1) CoPap shift in the direction opposite the postural disturbance (Fig. 3a); (2) CoPap shift in the same direction as the postural disturbance, to increase postural muscle activation on the side opposite the disturbance (Fig. 3b; and (3) without CoPap shift, a continuous increase in postural muscle activation toward S2 on the side opposite the disturbance (Fig. 3c). Prior to the start of CoPap shift in (1) and (2), increased TA activation or decreased triceps surae activation was observed with forward CoPap shift, and an increase in the triceps surae was observed with backward shift. The numbers of subjects showing postural preparation are shown in Table 2. Onset time of preparatory muscle activation was 645.8 ± 95.6 ms with forward translation and 662.2 ± 152.7 ms with backward translation. No significant difference in onset time was found between floor directions. With both floor directions, onset time of preparatory muscle activation was significantly later than P3 latency for all subjects showing postural preparation (time difference: forward 177.8 ± 64.6 ms (*t*_10_ = 9.13, *p* < 0.001); backward 210.5 ± 104.4 ms (*t*_10_ = 6.69, *p* < 0.001)), with no significant difference in time difference between floor directions. Significant correlations were found between onset time of preparatory muscle activation and P3 latency in both floor directions (forward: *r* = 0.81, backward: *r* = 0.74, *p* < 0.01).

**Table 2:** Number of subjects showing each type of postural preparation between S1 and S2 in the choice-reaction task

Types of postural preparation	Forward translation	Backward translation
CoPap shift in the direction opposite the postural disturbance	9	7
CoPap shift in the same direction as the postural disturbance	1	2
Continuous EMG increase without CoPap shift	1	2

*CoPap* center of pressure in the anteroposterior direction, *EMG* electromyogram

## Discussion

The present study set the choice-reaction task with the direction of floor translation indicated by S1 and simple-reaction tasks with fixed floor directions. No significant differences were apparent between these tasks in the time or amplitude of the CNV peak just before S2, and CoPap displaced positions or any postural muscle activation patterns after S2. The late CNV just before S2 is believed to reflect motor preparation processing and anticipatory attention directed to S2 [10, 11]. These results suggest that even under the choice-reaction task, postural preparation for the disturbance caused by S2 could be sufficient at the S2 point, resulting in the same postural control for the disturbance as in the simple-reaction task. We will mainly discuss the time relationship between attentional switching and postural preparation in the S1–S2 period in the choice-reaction task.

P3 component was clearly evident at about 450 ms in the choice-reaction task, but not in the simple-reaction task. As mentioned in the “Background” section, the latter part of P3 is considered to reflect attentional switching to S2 after S1 judgment in the S1–S2 choice-reaction paradigm with finger flexion [12–15]. In this study, S2 was the floor translation, and the reaction task to S2 was not voluntary movement. However, the P3 waveform in this study resembled that in previous studies with finger reaction [12–15]. S1 would be important to control standing posture appropriately against the perturbation of S2. Therefore, the attention would be initially directed to S1 and would then have to be switched to the upcoming process for postural disturbance.

Postural preparation in the S1–S2 period was found as CoPap shift preceded by the change of postural muscle activation and/or continuous EMG increase. The CoPap shift in the direction opposite that of the postural disturbance would relate to the control of standing position to moderate the influence of the disturbance. The continuous EMG increase toward S2, with or without CoPap shift in the same direction, would reflect the increase in muscle stiffness against the disturbance [7]. These postural preparations started about 180 ms (forward floor translation) or 210 ms (backward floor translation) after P3 peak. High positive correlations between P3 peak latency and time of onset of postural preparation were found among all subjects, regardless of disturbance direction. These results suggest that postural preparation for S2 would be started after attentional switching. In addition, no significant differences were seen between forward and backward translations in P3 latency, time of onset of postural preparation and time difference between them, suggesting that the timing of attentional switching or postural preparation would remain unchanged by the pitch of the S1 auditory sound or the selection of postural muscles against S2 direction. The P3 waveform and onset timing of anticipatory activity of postural muscles have been reported to change with time pressure on the process of attentional switching [5]. In a future study, a shorter S1–S2 interval (i.e., higher time pressure) using this same paradigm should allow clarification of the temporal relationship between attentional switching and postural preparation.

## Conclusions

In the S1–S2 choice-reaction paradigm with transient forward or backward floor translation (S2), with the direction indicated by S1, postural preparation for S2 was demonstrated to be started after attentional switching from S1 to S2, for which starting times were indicated by EMG change and P3 peak, respectively.

## Abbreviations

A/D: Analog-to-digital
BF: Biceps femoris
CNV: Contingent negative variation
CoPap: Center of pressure in the anteroposterior direction
EBL: Extreme backward leaning
EEG: Electroencephalogram
EFL: Extreme forward leaning
EMG: Electromyogram
EOG: Electrooculogram
ERP: Event-related brain potential
ES: Erector spinae
FL: Foot length
GcM: Medial head of gastrocnemius
QS: Quiet standing
RA: Rectus abdominis
RF: Rectus femoris
SD: Standard deviation
Sol: Soleus
TA: Tibialis anterior
